# Capturing intermediate filament networks

**DOI:** 10.7554/eLife.78248

**Published:** 2022-04-04

**Authors:** Pierre A Coulombe

**Affiliations:** 1 Department of Cell and Developmental Biology, Department of Dermatology, and Rogel Cancer Center, University of Michigan Medical School Ann Arbor United States

**Keywords:** cytoskeleton, intermediate filament, keratin, retinal pigment epithelium, 3D network, virtual reality, epithelia, Human, Mouse, Dog

## Abstract

Mapping intermediate filaments in three dimensions reveals that the organization of these filaments differs across cell types.

**Related research article** Windoffer R, Schwarz N, Yoon S, Piskova T, Scholkemper M, Stegmaier J, Bönsch A, Di Russo J, Leube R. 2022. Quantitative mapping of keratin networks in 3D. *eLife* **11**:e75894. doi: 10.7554/eLife.75894.

Cells are highly structured systems that contain a network of filaments known as the cytoskeleton. This scaffolding structure helps cells to maintain their shape, internal organization, and integrity under stress. It also plays important roles in intracellular transport, cell signaling and in cell proliferation, growth, differentiation and death. To fulfill their purpose, cytoskeletal filaments must be spatially organized, regulated and integrated in a manner that meets the rapidly changing needs of a cell (Kim and Coulombe, 2007; Block et al., 2015).

Recent advances in microscopy and image analysis have provided transformative insight into the three-dimensional (3D) architecture of cellular components in tissues, organs and entire organisms. Now, in eLife, Rudolf Leube and colleagues at RWTH Aachen University – including Reinhard Windoffer as first author – report on new insights into networks of intermediate filaments in three different types of epithelial cells (Windoffer et al., 2022).

The researchers used confocal microscopy and newly developed image analysis tools to describe the 3D organization of the entire intermediate filament network in three types of epithelial cells: MDCK cells, which are derived from canine kidneys, HaCaT keratinocytes derived from human skin, and retinal pigment epithelial (RPE) cells from mice. To visualize the network of keratin intermediate filaments in all three cell types, a specific keratin, known as Keratin 8 (K8), was tagged with a green fluorescence marker. The 3D models were generated based on microscopy images of filaments containing fluorescent K8, and the digitized representations of these images were analyzed at different scales to quantitatively describe the properties of the filaments and the networks they form.

From a methodology standpoint alone, the study by Windoffer et al. breaks new ground and sets the stage for an atlas-type collection holding information on the organization and architecture of intermediate filaments for a plethora of cell types, under various biological conditions. Among the myriad findings reported in this article, three stand out because they help capture the breadth of the study.

First, on a cellular scale, intermediate filaments show a specific spatial organization in the three cell types analyzed. For instance, MDCK kidney cells feature distinct apical and basal keratin intermediate filament networks that are interconnected but each possess unique features (Figure 1). HaCat cells are comparatively very flat and are densely packed with keratin filaments that enclose the nucleus laterally and include long bundles that run parallel to the cell’s longest axis. Retinal pigment epithelial (RPE) cells, which were analyzed in the natural context of the eye in situ, exhibit a comparatively less dense keratin intermediate filament network that is surprisingly prominent in the cytoplasmic apical domain, which faces photoreceptor cells in the retina. The mechanisms underlying these differences, and others reported by the researchers, are certainly worth investigating.

**Figure 1.**
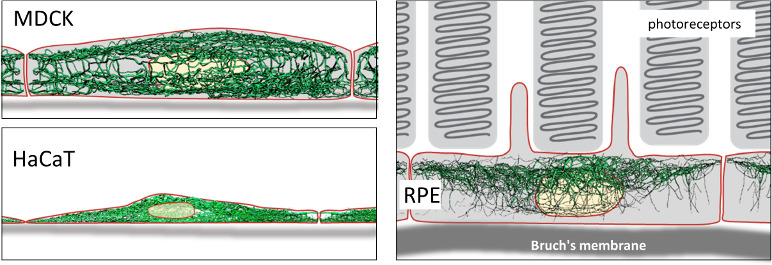
Intermediate filaments in different cell types. Top left: Lateral view of intermediate filament networks in canine kidney epithelial cells (MDCK). The connected apical and basal networks each possess unique features. Bottom left: Human skin keratinocytes (HaCaT) are flat and densely packed, with long bundles of intermediate filaments enclosing the nucleus laterally. Right: Retinal pigment epithelial cells (RPE) in the (mouse) eye in situ show a comparatively less dense network that is surprisingly prominent in the apical area, which faces photoreceptor cells in the retina.

Image credit: Images reproduced from Windoffer et al. with permission, CC BY 4.0 (Windoffer et al., 2022).

Second, at a subcellular level, Windoffer et al. provide a thorough account of several key properties of intermediate filaments, which help inform the reciprocal interplay between mechanical forces and network architecture at both local and cell-wide levels. Again, an interesting mix of convergence and divergence are reported for the three cell types analyzed.

Third, at a molecular level, conversions of digital representations of the intermediate filaments into biochemical quantities revealed that the mass of keratin in skin keratinocytes is similar to mass measurements obtained using quantitative western blotting (Feng et al., 2013). Such detailed knowledge can help guide future efforts to identify and characterize the mechanisms that regulate the amount of keratin proteins and filaments present in several types of epithelial cells.

With this rigorous, innovative and elegant study, Windoffer et al. provide a methodological framework to probe the architecture and organizing principles of intermediate filament networks for any cell type in 3D. This contribution is timely in that it complements emerging evidence about the high-resolution structure of mature, intermediate filaments emanating from cryo-electron tomography imaging (e.g., Turgay et al., 2017; Weber et al., 2021) and also from crystallographic studies (e.g., Lee et al., 2020; Eldirany et al., 2021).

High resolution information about both core architecture of individual intermediate filaments and their spatial organization as intricate 3D networks within cells is sorely needed to foster a deeper understanding of their mechanical and non-mechanical roles in epithelial cells, and their disruption in disease. As we await additional quantitative data of intermediate filaments networks in other cell types and/or biological circumstances, the study by Windoffer et al. sets the stage for follow-up analyzes regarding the principles and mechanisms underlying the spatial organization of intermediate filaments in vivo.

## References

[bib1] Block J, Schroeder V, Pawelzyk P, Willenbacher N, Köster S (2015). Physical properties of cytoplasmic intermediate filaments. Biochimica et Biophysica Acta.

[bib2] Eldirany SA, Lomakin IB, Ho M, Bunick CG (2021). Recent insight into intermediate filament structure. Current Opinion in Cell Biology.

[bib3] Feng X, Zhang H, Margolick JB, Coulombe PA (2013). Keratin intracellular concentration revisited: implications for keratin function in surface epithelia. The Journal of Investigative Dermatology.

[bib4] Kim S, Coulombe PA (2007). Intermediate filament scaffolds fulfill mechanical, organizational, and signaling functions in the cytoplasm. Genes & Development.

[bib5] Lee CH, Kim MS, Li S, Leahy DJ, Coulombe PA (2020). Structure-function analyses of a keratin heterotypic complex identify specific keratin regions involved in intermediate filament assembly. Structure (London, England: 1993).

[bib6] Turgay Y, Eibauer M, Goldman AE, Shimi T, Khayat M, Ben-Harush K, Dubrovsky-Gaupp A, Sapra KT, Goldman RD, Medalia O (2017). The molecular architecture of lamins in somatic cells. Nature.

[bib7] Weber MS, Eibauer M, Sivagurunathan S, Magin TM, Goldman RD, Medalia O (2021). Structural heterogeneity of cellular K5/K14 filaments as revealed by cryo-electron microscopy. eLife.

[bib8] Windoffer R, Schwarz N, Yoon S, Piskova T, Scholkemper M, Stegmaier J, Bönsch A, Di Russo J, Leube R (2022). Quantitative mapping of keratin networks in 3D. eLife.

